# Case Report: Lennox–Gastaut Epileptic Encephalopathy Responsive to Cannabidiol Treatment Associated With a Novel *de novo* Mosaic *SHANK1* Variant

**DOI:** 10.3389/fgene.2021.735292

**Published:** 2021-11-29

**Authors:** Justyna Paprocka, Szymon Ziętkiewicz, Joanna Kosińska, Ewa Kaczorowska, Rafał Płoski

**Affiliations:** ^1^ Department of Pediatric Neurologsluy, Faculty of Medical Science in Katowice, Medical University of Silesia, Katowice, Poland; ^2^ Department of Medical Genetics, Medical University of Warsaw, Warsaw, Poland; ^3^ Intercollegiate Faculty of Biotechnology, University of Gdansk, Gdańsk, Poland; ^4^ Department of Biology and Medical Genetics, Medical University of Gdańsk, Gdańsk, Poland

**Keywords:** epilepsy, Lennox–Gastaut syndrome, Rap1, treatment, SHANK1

## Abstract

The SH3 and multiple ankyrin repeat domains (SHANKs) are a family of scaffolding proteins located in excitatory synapses required for their development and function. Molecular defects of SHANK3 are a well-known cause of several neurodevelopmental entities, in particular autism spectrum disorders and epilepsy, whereas relatively little is known about disease associations of SHANK1. Here, we propose a novel *de novo* mosaic p.(Gly126Arg) *SHANK1* variant as the monogenic cause of disease in a patient who presented, from the age of 2 years, moderate intellectual disability, autism, and refractory epilepsy of the Lennox–Gastaut type. The epilepsy responded remarkably well to cannabidiol add-on therapy. *In silico* analyses including homology modeling and molecular dynamics simulations indicated the deleterious effect of SHANK1 p.(Gly126Arg) on the protein structure and the related function associated with protein–protein interactions. In particular, the variant was predicted to disrupt a hitherto unknown conserved region of SHANK1 protein with high homology to a recently recognized functionally relevant domain in SHANK3 implicated in ligand binding, including the “non-canonical” binding of Rap1.

## Introduction

Lennox–Gastaut syndrome (LGS; ORPHA:2382) is an epileptic encephalopathy refractory to treatment characterized by three major features: polymorphic seizures, intellectual impairment, and characteristic electroencephalogram (EEG) pattern (Asadi-Pooya, 2018; Auvin, 2020). Although no causative genes have been identified in cases without a structural brain abnormality, *de novo* mutations in *ST3GAL3*, OMIM#615006; *YWHAG*, OMIM#617665; or *KCNT2*, OMIM#617771 have been reported.

**FIGURE 1:**
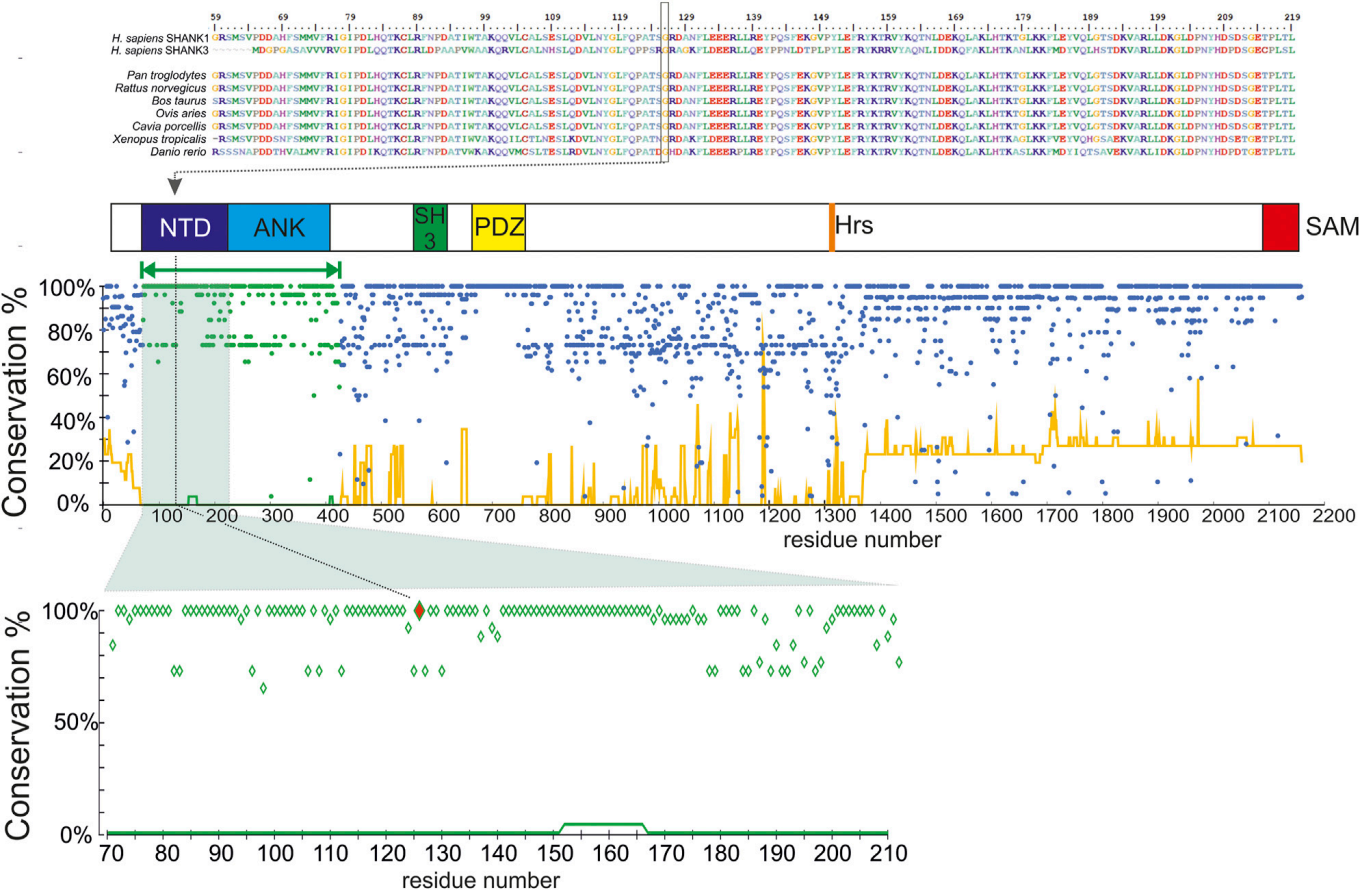
Domain organization and evolutionary conservation of the SHANK1 protein. Top, the alignment of N-terminal regions of *H. sapiens* SHANK1 and SHANK3 proteins, and SHANK1 homologs from selected species (N = 7, selected for maximal divergence), and the mutation site (Gly126) is indicated by a box and a dotted line. Below, the domain organization of SHANK1 protein (NTD, N-terminal domain; ANK, ankyrin repeat region, SH3 domain; HRS, Homer recognition site; SAM, sterile alpha motif (oligomerization site). Homology modeled region marked by green bar. Bottom, the conservation plot for the *H. sapiens* SHANK1 protein. Dots, % of residue identical to *H. sapiens* (excluding deletions) at a given position in the multiple sequence alignment of homologous proteins (N = 25), line, % of deletions at a given position in the same alignment. Green dots/lines denote the modeled region. Below, a close—in view of the conservation plot for the N-terminal domain fragment—mutation site (Gly126) marked in red.

In the human genome, there are three genes (*SHANK1-3*) coding for SH3 and multiple ankyrin repeat domain proteins (SHANK): The three SHANK proteins constitute the main component of postsynaptic density of glutamatergic synapses of the central nervous system. Although the SHANKs (also called proline-rich synapse-associated proteins, ProSAP) vary in tissue expression patterns, they all share similar domain composition and function (Monteiro and Feng, 2017). They comprise the N-terminal ankyrin repeat region, followed by SH3 and PDZ domains. Next, following a long proline-rich region, a SAM domain is located at the C terminus (Figure 1). The molecular role for all SHANK proteins is the formation of the structural scaffold connecting and organizing numerous synaptic proteins. While the SAM domain contributes to SHANK oligomerization, the remaining regions bind, directly or indirectly, to a broad range of interactors including actin-based cytoskeleton, membrane receptors (Jiang and Ehlers 2013; Monteiro and Feng 2017), and the Ras/Rap small GTPases which bind to the N-terminal domain preceding the ANK repeat region (Lilja et al., 2017). For SHANK3, an additional binding site for these GTPases formed between N-terminal domain and ANK repeats was demonstrated (Cai et al., 2020).

Molecular defects in the SHANK family have been linked to a range of neurodevelopmental and psychiatric disorders (Leblond et al., 2014; de Sena Cortabitarte et al., 2017; Monteiro and Feng, 2017; Berg et al., 2018). *SHANK3* is the most recognized member of the family, being associated with the Phelan–McDermid syndrome (OMIM#606232). SHANK1 connects receptors of the postsynaptic membrane including the NMDA-type and metabotropic glutamate receptors with the actin-based cytoskeleton via the Homer and GKAP/PSD-95 complexes (Leblond et al., 2014; de Sena Cortabitarte et al., 2017; Monteiro and Feng, 2017; Berg et al., 2018). Based on the general role of SHANK family proteins, it has been suggested that *SHANK1* defects might also be involved in the pathogenesis of neurodevelopmental disorders (Leblond et al., 2014; de Sena Cortabitarte et al., 2017; Monteiro and Feng, 2017; Berg et al., 2018).

The analysis of *SHANK1* in the GnomAD population shows that despite considerable polymorphism, there is an underrepresentation of both loss-of-function (LOF) variants (observed/expected (o/e) = 0.01, 90% CI 0.08–0.18) and missense variants (o/e = 0.69, 90% CI 0.65–0.73) (https://gnomad.broadinstitute.org).

To date, ∼30 patients with *SHANK1* defects have been reported (the Human Gene Mutation Database HGMD 2019.4; DECIPHER (Firth et al., 2009) accessed February 2021). These included main families with small (63.4 kb *de novo* and 63.8 kb inherited) deletions reported by Sato et al. (2012), which were considered causes for autism spectrum disorders (ASD) in a male sex-dependent penetrance model. The study however did not exclude an alternative diagnosis as whole-exome sequencing (WES) was not performed (Sato et al., 2012). Other reported copy number variants (CNVs) are much larger, encompassing several recognized morbid genes; hence, the effect of *SHANK1* remains indeterminate. Among single-nucleotide variants (SNVs) associated with developmental delay, ASD or schizophrenia, only five were *de novo* (Firth et al., 2009; Fromer et al., 2014; Wang et al., 2016; Kosmicki et al., 2017), whereas the remaining were inherited, suggesting that they had low penetrance or were neutral (Guo et al., 2018). However, the pathogenicity of the *de novo* variants was not clear since functional studies were not performed (Firth et al., 2009; Fromer et al., 2014; Wang et al., 2016; Kosmicki et al., 2017). Recently, May at al. reported six patients with a diverse spectrum of neurodevelopmental disorders associated with truncating *SHANK1* variants, two of which were studied functionally (May et al., 2021). Here, we describe the first patient with autism and epileptic encephalopathy (Lennox–Gastaut syndrome) and a *de novo* mosaic *SHANK1* variant which according to *in silico* modeling disrupts a predicted hitherto unknown functionally important region of SHANK1.

## Case Presentation

An 11-year-old boy with Lennox–Gastaut syndrome, global developmental delay, and autistic features without congenital defects or facial dysmorphic features and negative family history for neurodevelopmental disorders was referred for WES. The gestation and delivery period were uneventful. The first epileptic seizures appeared at the age of 2 years. At that time, there was also profound hypotonia with preserved tendon reflexes. The boy was not able to walk independently or communicate verbally. The EEG showed generalized paroxysmal changes with slow spike-and-wave complexes (<3 Hz, Supplementary Figure S1). Brain MRI and neurometabolic workup (tests for organic acids in urine, acylcarnitine profile, biotinidase activity, transferrin isoforms, amino acid profiles in blood and CSF, very long–chain fatty acid (VLCFA) levels, screening for lysosomal storage disorders, and microarray comparative genomic hybridization (aCGH) were normal. Polymorphic seizures (tonic–clonic, atonic, myoclonic, and absence seizures) required numerous changes of therapy. The drug combinations used were as follows: valproic acid + vigabatrin, valproic acid + lamotrigine, valproic acid + lamotrigine + phenobarbital, valproic acid + topiramate, valproic acid + topiramate + clonazepam, valproic acid + levetiracetam, and valproic acid + levetiracetam + clobazam. The antiepileptic drugs were given in maximal doses. At the age of 7 years, he was started on treatment with cannabidiol (30 mg/kg/day) in a clinical trial (an open-label extension study to investigate the safety of cannabidiol (GWP42003-P; CBD) in children and adults with inadequately controlled Dravet or Lennox–Gastaut syndrome (2014-001834-27, finished: Sep 24, 2020). The frequency of seizures before the cannabidiol therapy was up to twenty a day. Status epilepticus occurred twice with good response to Relanium and clonazepam intravenously. After cannabidiol administration, the epileptic seizures stopped, and the patient has been seizure-free for >4 years now. Currently, the patient is receiving levetiracetam and cannabidiol only. The most recent neurological examination showed the head circumference of 52 cm (10th centile) and generalized hypotonia with preserved tendon reflexes. On psychological evaluation, moderate intellectual disability and autism spectrum disorder were found. He counts to 10, knows a few letters, and follows simple commands. He is systematically rehabilitated due to hypotonia and posture defects. The most recent EEG showed minor abnormalities with generalized medium-amplitude slow waves, rarely accompanied with sharp waves.

## Materials and Methods

Blood samples were collected from the proband, his parents, and his sister. WES was initially performed on the proband’s DNA sample using the Twist Human Core Exome (Twist Bioscience, San Francisco, CA, United States) and paired-end sequencing (2x100 bp) on NovaSeq 6000 (Illumina). The mean coverage was 96x, 99.9% of target was covered ≥10x, and 99.4% was covered ≥20x. The analysis of the WES data and variant prioritization were performed, as previously described (Ploski et al., 2014), except that the Hg38 human genome version was used for alignment. Variant validation and family studies were performed by amplicon deep sequencing (ADS) using the Nextera XT kit (Illumina) and NovaSeq 6000 (Illumina). The novel *SHANK1* variant has been submitted to the Leiden Open Variation Database (https://databases.lovd.nl/shared/screenings/0000376764) (Fokkema et al., 2011). *In silico* evaluation of the SHANK1 Gly126Arg variant is described in supplementary material. Subsequently, to rigorously exclude the contribution of other variants to the proband’s phenotype, WES was also performed in his parents (using the same methods). The mean coverage, ≥10x and ≥20x coverage was 80x, 99.6%, 99.3% and 96x, 99.7%, 99.4%, for the father and mother, respectively.

## Results

### Genetic Study

**FIGURE 2:**
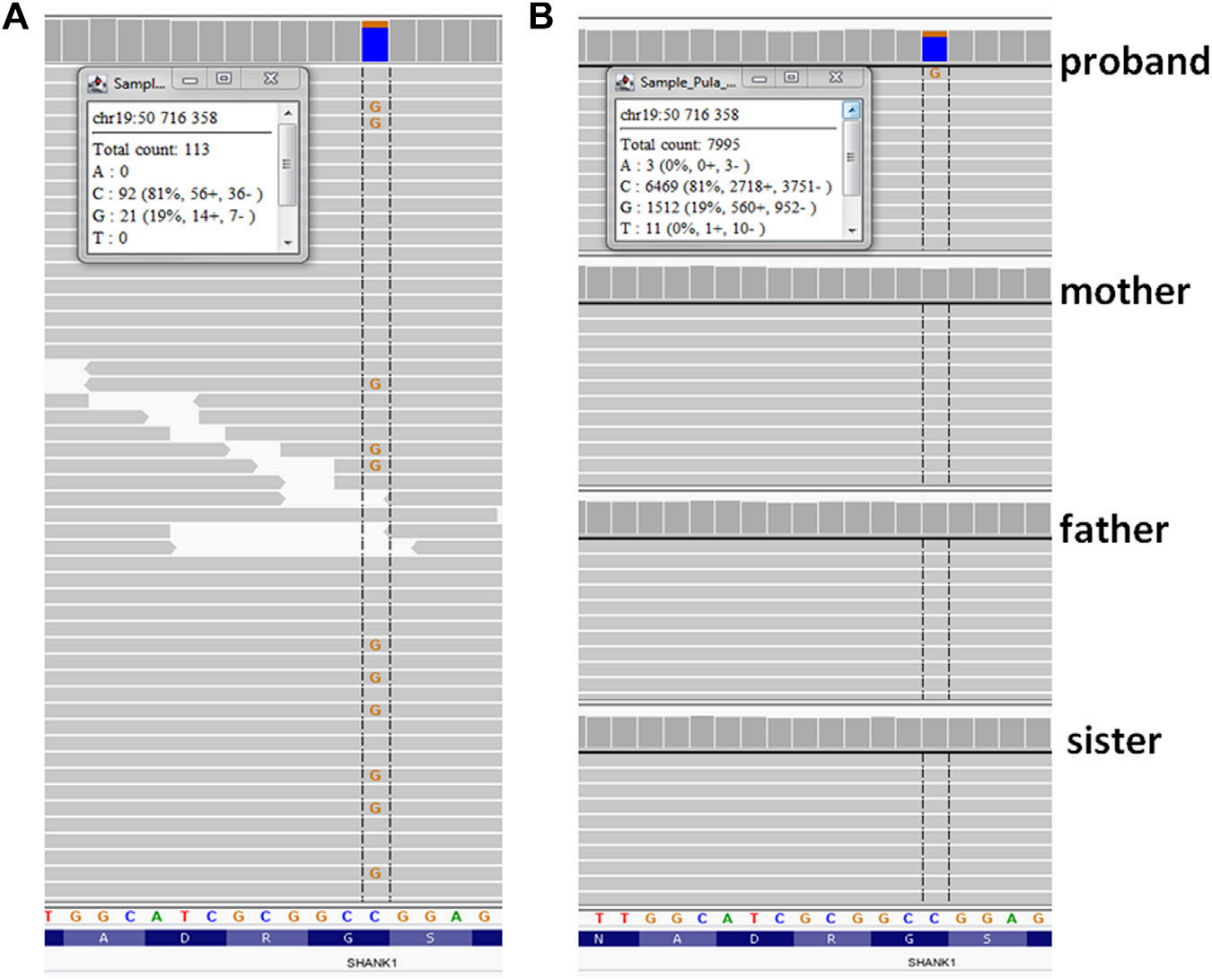
Results of sequencing variant identified in *SHANK1*gene—Integrative Genomic Viewer (IGV) screen shot. **(A)** whole-exome sequencing; **(B)** ADS (amplicon deep sequencing). Insets show the numbers and percentages of variant NGS reads.

A heterozygous variant in the *SHANK1* gene [hg38; chr19:g.050716358-C > G, NM_016148.5: c.376 = /G > C, p.(Gly126Arg)] (Figure 2) was found on 21% of all reads (N = 113/X), suggesting it was mosaic. Co-segregation studies using ADS confirmed the variant as *de novo* and showed mosaicism at the level of 19% of reads (N = 7995). The variant was absent from all population databases tested (gnomAD, Bravo, and an in-house database of >3000 WES of Polish individuals), and it was not listed in ClinVar or HGMD v2019.4. According to the ACMG 2015 criteria, the variant was classified as variant of unknown significance (VUS; PM2: absent from controls, PP3: *in silico* evidence, and BP1: known *SHANK1* missense variants are predominantly benign). The list of all variants considered in the proband is given in Supplementary Table S1.

### *In silico* Evaluation of the SHANK1 Variant

Sequence alignment showed that the Gly126 is the residue 100% conserved among homologous proteins from various species as well as in human SHANK3 (Figure 1). Furthermore, multiple alignments of human SHANK1 and its 25 homologs confirmed that the N-terminal domain of SHANK1, where Gly126 is located, is the most conserved part of the protein. According to our analysis, this region (defined as aa71-425) contains 54% of fully conserved aa, whereas for other domains, the complete (i.e., 100%) conservation was observed for a minority of residues (15%). Also, among SHANK1 orthologs, the occurrence of deletions relative to the *H. sapiens* sequence was clearly lower in this region (Figure 1).

**FIGURE 3:**
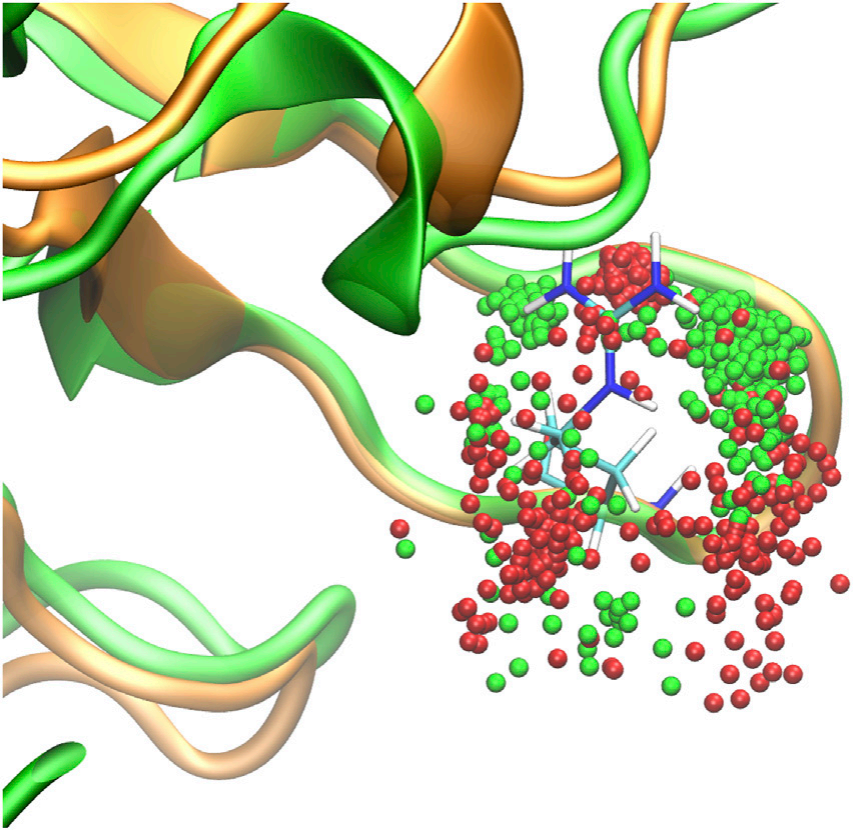
Molecular dynamics trajectory of SHANK1 WT (green backbone) and Gly126Arg (orange backbone). Dots represent 0.5 ps positions of Cζ of Arg127: green dots for WT trajectory and red dots for the Gly126Arg variant. The starting position of WT Arg127 is shown in stick representation.

The molecular modeling showed that in the wild-type (WT) SHANK1, residues 124 to 127 constitute a typical class I turn, with i:i + 4 hydrogen bond formed between Thr124 and Arg127 (Figure 3). When comparing the 200 ns molecular dynamics (MD) trajectories for WT and mutant proteins, we found that the backbone hydrogen bonds between Thr124 and Arg127, which were present in 23.3% (Thr124 as the donor) and 67.4% (Thr124 as the acceptor) of the simulation time, were disrupted in the Gly126Arg variant, being present merely for 4.2 and 26.3% of time. In addition, in the presence of the Gly126Arg variant, the adjacent Arg127 was shifted to the “outward” conformation for obtaining more simulation time than the WT. The only “inward” cluster of Arg127 positions corresponded to the side chain conformation in which its quaternary amine participated in hydrogen bond formation with the Thr124 backbone (30.1% of simulation time), substituting the WT-like backbone–backbone H-bond, a position encountered during WT simulation for only 1.7% of time.

We have also constructed a model of hypothetical WT SHANK1–Rap1 (GTP) complex, assuming the non-canonical lower affinity binding site, as described for SHANK3 (Cai et al., 2020) (Supplementary Figure S2). Intriguingly, in the place of the salt bridge Lys66 (SHANK3)–Asp38 (Rap1), described as important for SHANK3–Rap1 interaction (Cai et al., 2020) and not possible in SHANK1 as the corresponding residue Asn130 was not bearing a positive charge, SHANK1 was observed to easily establish a neighboring Arg127 (SHANK1)–Asp38 (Rap1) persistent salt bridge instead (Supplementary Figure S2). However, for establishing this interaction, Arg127 is required to exist in the “inward” conformation before docking the Rap1 molecule. This conformation, as shown before, is the most frequent one for the WT SHANK1 model but occurs less frequently in Gly126Arg variant model. Therefore, the mutation of SHANK1 Gly126 may effectively disrupt the modeled Rap1-binding region by shifting adjacent Arg127 orientation. Additionally, arginine substitution at this locus results in two neighboring arginines, which increases positive charge and additionally destabilizes the putative Shank1–Rap1 interaction electrostatically.

## Discussion

The *SHANK1* heterozygous p.Gly126Arg variant found in our patient is a previously unreported missense mutation which occurred *de novo*, most likely as an early postzygotic event present in approximately 40% of mature cells. The p.Gly126 is located in the evolutionary conserved N-terminal domain of SHANK1 protein and is extremely conserved being present in all identified 25 SHANK1 homologs.

Results of molecular modeling for WT SHANK1 show that the mutated Gly126 lies at an apex of the hairpin turn. For such tight turns, at the i + 2 position, that is, SHANK1Gly126, the glycine residue is encountered most frequently because of its high conformational freedom. Substitution with a large residue, like Gly126Arg, would lead to steric hindrance and destabilization of this structural motif. Also, electrostatic effects arising from arginine positive charge may further disrupt this region.

Indeed, our MD simulation showed that Gly126Arg significantly disrupted the formation of the hydrogen bonds between Thr124 and Arg127—these were present for 90.7% (23.3 + 67.4) of the time in the WT protein but only for 30.5% (4.2 + 26.3) of time in the mutant. The lack of the Thr124–Arg127 hydrogen bonds led to Arg127 shift to the “outward” conformation, which considerably affected the local SHANK1 structure.

SHANK1 and 3 possess a conserved Ras/Rap recognition site for GTP-bound small GTPases from Ras/Rap family in their N-terminal domains. Lilja et al. reported that both proteins sequester Rap1 and R-Ras *in vivo* (Lilja et al., 2017). Interestingly, recent crystallographic and biochemical analyses by Cai et al. showed that SHANK3 has additional atypical Ras/Rap binding region, formed between the ANK repeat and NTD domains (Cai et al., 2020). Whereas Cai et al. postulated that SHANK1 does not bind RAP1 in a similar place, one argument was that the non-canonical SHANK3–RAP1 binding depended on two pairs of salt bridges: Lys (SHANK3)–Asp38 (RAP1) and Arg72 (SHANK3)–Glu62 (RAP1) (Cai et al., 2020). In SHANK1, relative to the SHANK3–RAP1 interface, the only interaction missing is caused by the substitution of charged Lys66 SHANK3 with neutral Asn130. However, our modeling suggests that in the WT SHANK1, this interaction can be rescued by the nearby Arg127 side chain (although we emphasize that this should be confirmed experimentally). Interestingly, as discussed earlier, our modeling indicates that Arg127 in the mutated SHANK1 is displaced by the local destabilizing effect of Gly126Arg SHANK1, which removes the interaction with RAP1. The disruptive effect of Gly126Arg on the putative SHANK1–RAP1 non-canonical binding was further confirmed by the observation that in addition to the effect on conformation of Arg127, the important difference between the WT and Gly126Arg SHANK1 in the Rap1 interaction was the repositioning of the mutated Arg126.

Whereas the mechanism of pathogenicity for SHANK1 Gly126Arg variant remains to be directly demonstrated, the disruption of Rap1 [and/or other Ras/Rap protein(s)] binding is an intriguing hypothesis, given the good response of the proband’s epilepsy to cannabidiol. Whereas cannabinoid therapy has recently been recommended as an add-on epilepsy treatment of Lennox–Gastaut and Dravet syndromes, the precise subset of patients likely to respond remains undefined (Arzimanoglou et al., 2020). As cannabinoid signaling modulates Rap1 activity (Rueda et al., 2002; He et al., 2005), it is intriguing to speculate that the presence of Rap1 dysregulation in a patient could be helpful for predicting their clinical response to cannabidiol. This theory would also be consistent with the recent finding that pathogenic variants in Ras/Rap GTPase-activating protein SynGAP, which acts on RAP1 (Krapivinsky et al., 2004), also cause epilepsy alleviated by cannabidiol (Kuchenbuch et al., 2020).

The functional relevance of the “non-canonical” GTPase binding of SHANK3 (and thus, indirectly, the relevance of the putative SHANK1 motive encompassing Gly126) is further supported by the data on a SHANK3 variant in this region, the Leu68Pro (corresponding to SHANK1 Leu132), which was associated with autism (Gauthier et al., 2009) and shown to affect SHANK3 interactions with GTPase and other ligands such as sharpin and α-fodrin (Mameza et al., 2013).

In conclusion, we report a case of Lennox–Gastaut epileptic encephalopathy responsive to cannabidiol treatment associated with a novel SHANK1 variant. This variant is predicted to disrupt a hitherto unknown conserved region of SHANK1 protein with high homology to a recently recognized functionally relevant domain in SHANK3 implicated in ligand binding, including the “non-canonical” binding of Rap1.

## Data Availability

The datasets presented in this study can be found in online repositories. The names of the repository/repositories and accession number(s) can be found below: https://databases.lovd.nl/shared/screenings/0000376764, 0000376764.
