# Supplementary material for: Case Report: Lennox–Gastaut Epileptic Encephalopathy Responsive to Cannabidiol Treatment Associated With a Novel de novo Mosaic SHANK1 Variant
Source: Front Genet. 2021 Nov 29;12:735292. doi: 10.3389/fgene.2021.735292 (PMC8667173; doi:10.3389/fgene.2021.735292)
Supplement: Supplementary file 1 [file DataSheet1.PDF]

Supplementary materials and methods

In silico evaluation of the SHANK1 Gly126Arg variant

For evolutionary conservation analysis, the SHANK1 homologs between 95 and 35% identity were identified by HMMER algorithm from Uniref90 database using ConSurf web server(Ashkenazy et al., 2016) yielding 25 unique sequences which were aligned to obtain residue conservation percentage.

The homology model for the N-terminal fragment (NTD-ANK, residues 71-425) of human SHANK1 was built using SwissModel server (Waterhouse et al., 2018) based on a crystal structure of rat SHANK3 protein 6kyk.pdb(chain A) (68.1% identity, 81% similarity). The Gly126Arg model was obtained by altering the side chain in DeepView(Guex and Peitsch, 1997). Molecular dynamics (MD) simulation was performed in GROMACS 2021 (Abraham et al., 2015)(Amber99SB-ILDN (Lindorff-Larsen et al., 2010) force field) for 200 ns. The last trajectory frame were energy minimized.

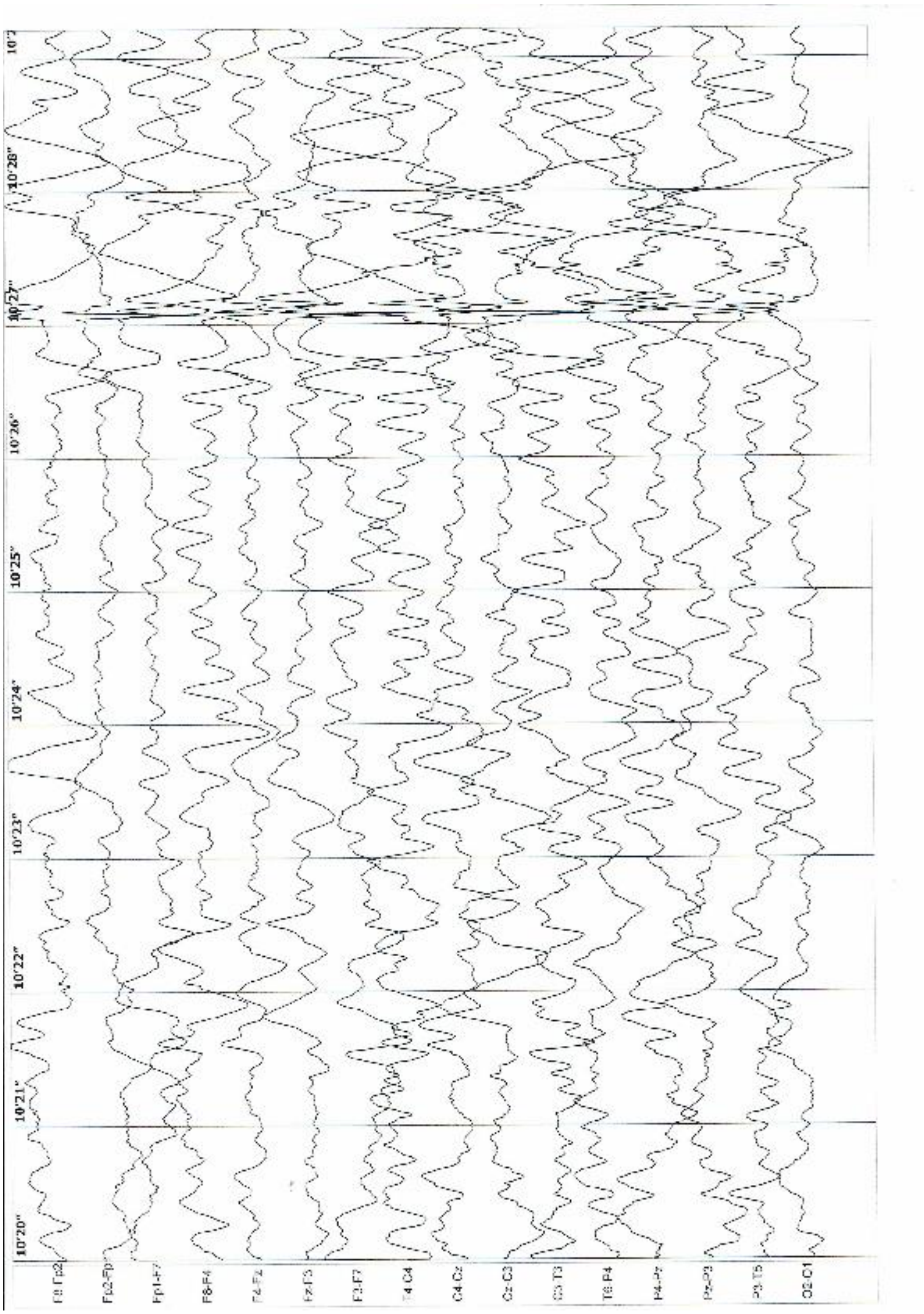

Supplemental figure 1 Paroxysmal changes in EEG tracing

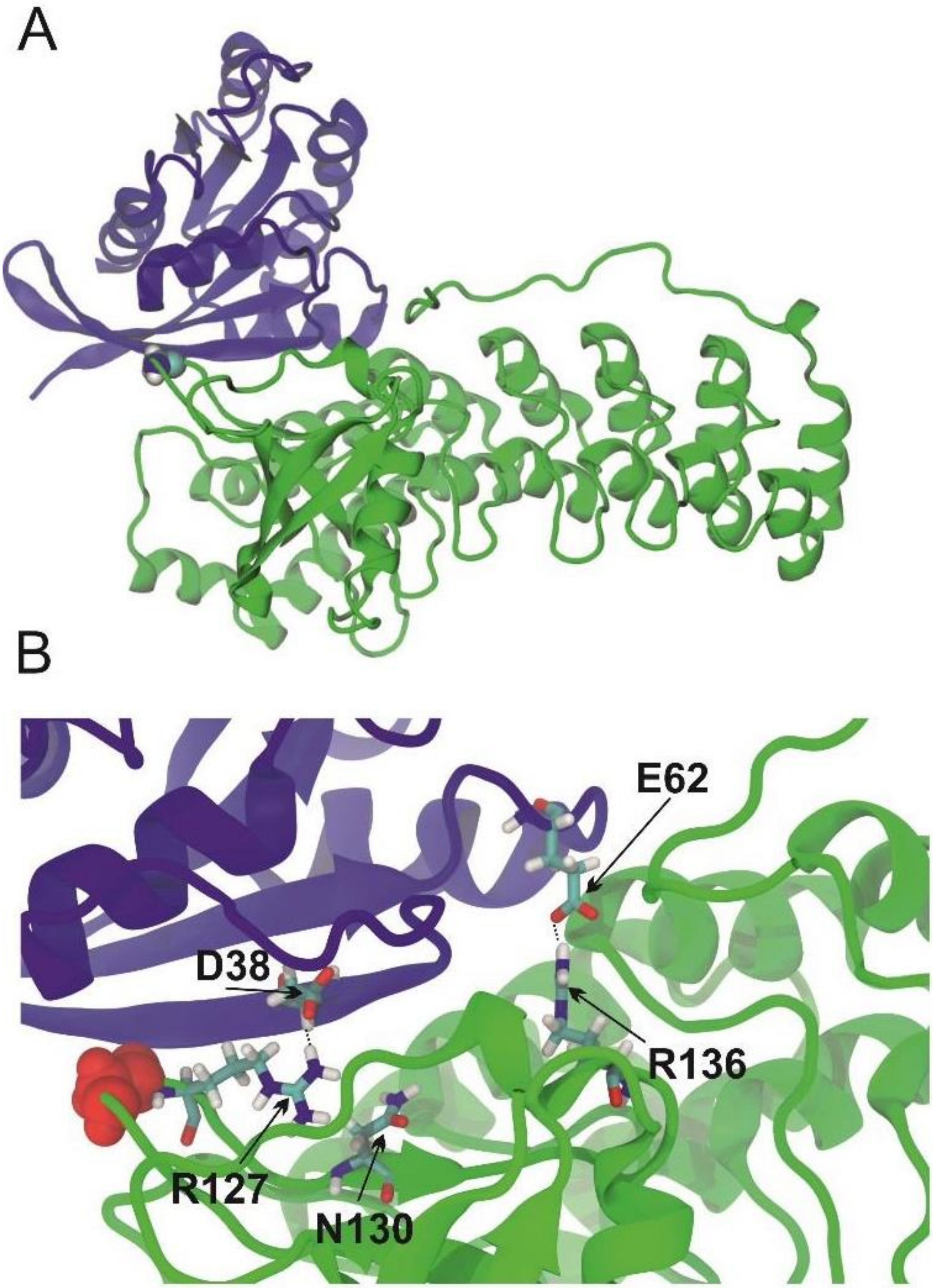

Supplementary figure 2 A) Model of putative SHANK1/Rap1 complex, 200ps frame from MD trajectory. Green, SHANK1, with Gly125 in ball representation. Blue, Rap1. B) Close view on putative SHANK1-Rap1 interface. The Gly126 in red. Stick representations show the residues forming intermolecular salt bridges. The SHANK1/Rap1 pair Arg136-Glu62 corresponds to SHANK3/Rap1 pair Arg72-Glu62. Instead of the SHANK3/Rap1 pair Lys66 (corresponding to SHANK1 uncharged Asn130) – Asp38, the Arg127-Asp38pair is formed between SHANK1 and Rap1.

Supplementary references

Abraham, M.J., Murtola, T., Schulz, R., Páll, S., Smith, J.C., Hess, B., et al. (2015). GROMACS: High performance molecular simulations through multi-level parallelism from laptops to supercomputers. SoftwareX 1, 19-25.

Ashkenazy, H., Abadi, S., Martz, E., Chay, O., Mayrose, I., Pupko, T., et al. (2016). ConSurf 2016: an improved methodology to estimate and visualize evolutionary conservation in macromolecules. Nucleic Acids Res 44(W1), W344-350. doi: 10.1093/nar/gkw408.

Guex, N., and Peitsch, M.C. (1997). SWISS-MODEL and the Swiss-PdbViewer: an environment for comparative protein modeling. Electrophoresis 18(15), 2714-2723. doi: 10.1002/elps.1150181505.

Lindorff-Larsen, K., Piana, S., Palmo, K., Maragakis, P., Klepeis, J.L., Dror, R.O., et al. (2010). Improved side-chain torsion potentials for the Amber ff99SB protein force field. Proteins 78(8), 1950-1958. doi: 10.1002/prot.22711.

Waterhouse, A., Bertoni, M., Bienert, S., Studer, G., Tauriello, G., Gumienny, R., et al. (2018). SWISS-MODEL: homology modelling of protein structures and complexes. Nucleic Acids Res 46(W1), W296-W303. doi: 10.1093/nar/gky427.
